# IL-10- and TGFβ-mediated Th9 Responses in a Human Helminth Infection

**DOI:** 10.1371/journal.pntd.0004317

**Published:** 2016-01-05

**Authors:** Rajamanickam Anuradha, Saravanan Munisankar, Yukthi Bhootra, Jeeva Jagannathan, Chandrakumar Dolla, Paul Kumaran, Thomas B. Nutman, Subash Babu

**Affiliations:** 1 National Institutes of Health—NIRT—International Center for Excellence in Research, Chennai, India; 2 National Institute for Research in Tuberculosis, Chennai, India; 3 Laboratory of Parasitic Diseases, National Institutes of Allergy and Infectious Diseases, National Institutes of Health, Bethesda, Maryland, United States of America; The Ohio State University, UNITED STATES

## Abstract

**Background:**

Th9 cells are a subset of CD4^+^ T cells that express the protoypical cytokine, IL-9. Th9 cells are known to effect protective immunity in animal models of intestinal helminth infections. However, the role of Th9 cells in human intestinal helminth infections has never been examined.

**Methodology:**

To examine the role of Th9 cells in *Strongyloidis stercoralis* (Ss), a common intestinal helminth infection, we compared the frequency of Th9 expressing IL-9 either singly (mono-functional) or co-expressing IL-4 or IL-10 (dual-functional) in Ss-infected individuals (INF) to frequencies in uninfected (UN) individuals.

**Principal Findings:**

INF individuals exhibited a significant increase in the spontaneously expressed and/or antigen specific frequencies of both mono- and dual-functional Th9 cells as well as Th2 cells expressing IL-9 compared to UN. The differences in Th9 induction between INF and UN individuals was predominantly antigen-specific as the differences were no longer seen following control antigen or mitogen stimulation. In addition, the increased frequency of Th9 cells in response to parasite antigens was dependent on IL-10 and TGFx since neutralization of either of these cytokines resulted in diminished Th9 frequencies. Finally, following successful treatment of Ss infection, the frequencies of antigen-specific Th9 cells diminished in INF individuals, suggesting a role for the Th9 response in active Ss infection. Moreover, IL-9 levels in whole blood culture supernatants following Ss antigen stimulation were higher in INF compared to UN individuals.

**Conclusion:**

Thus, Ss infection is characterized by an IL-10- and TGFβ dependent expansion of Th9 cells, an expansion found to reversible by anti-helmintic treatment.

## Author Summary

*Strongyloides stercoralis* is a common intestinal parasite affecting about 50–100 million people worldwide. It is characterized by a complex lifecycle involving both free- living and parasitic stages and the clinical manifestations range from asymptomatic infection to multi-organ failure. It has the propensity to cause disseminated disease and death in immunocompromised individuals. Therefore, an in depth understanding of the immune responses to this helminth parasite is warranted. However, what we know about the immunity to this infection is mostly derived from animal studies. Th9 cells are a subset of CD4+ T cells producing the cytokine—IL-9. Since Th9 cells are increasingly recognized as being important in immunity to intestinal infection with helminths, we examined the induction and regulation Th9 cell responses to Ss infection utilizing infected and uninfected individuals from an endemic area in India. We show that Ss infection is characterized by profound alterations in the Th9 compartment and that this response is mainly regulated by the cytokines—IL-10 and TGFβ. In addition, we also demonstrate that active infection is a pre-requisite for this regulation and anti-Ss treatment can dampen enhanced Th9 responses.

## Introduction

Upon antigen-specific stimulation, CD4^+^ T cells have the potential to differentiate into various T-helper (Th) cell subsets based on the pattern of transcription factors induced and cytokines produced [1]. Traditionally associated with the Th2 response, IL-9 is a member of the common γ chain cytokine family and exerts broad effects on many cell types including mast cells, eosinophils, T cells and epithelial cells [2,3]. However, more recently, a CD4^+^ T cell subset with the exclusive capacity to secrete IL-9 has been described [4,5]. This CD4^+^ T cell subset is thought to develop under the influence of IL-4 and TGFβ and to produce IL-9, either singly or in conjunction with IL-10; these cells fail to produce IL-4 [6,7]. However, little data are available on the expression pattern of Th9 cells in humans.

Th9 cells in humans were initially described as IL-9^+^and IL-17^+^ [8]; however, IL-9 producing CD4^+^ T cells distinct from Th1, Th2 and Th17 cells have also been described [9,10]. Th9 cells, in humans, can play a protective (tumors [11]) as well as a pathogenic (allergy [12], atopy [13], asthma [12] and auto-immunity [14]) role in differing disease states. Although, Th9 cells have been implicated in resistance to intestinal helminth infection in animal models [5,15,16,17], the role of Th9 cells in human intestinal helminth infections has never been explored.

Human infections with *Strongyloides stercoralis* (Ss) appears to be controlled by a Th2 response [18,19,20]. Moreover, protective immunity to Ss larvae in mice is dependent on CD4^+^ T cells, and these cells typically produce IL-4 and IL-5 [21]. Finally, primary infections of rats or mice with the rodent parasites, *S*. *ratti* and *S*. *venezuelensis* respectively, results in a Th2 response, with production of IL-4, IL-5 and IL-13 and concomitant suppression of IFNγ [22]. We have previously demonstrated that Ss infection is associated with down regulation of parasite-antigen specific Th1 and Th17 responses and up regulation of parasite-antigen specific Th2 responses [23].

Therefore, we sought to determine the regulation of Th9 cells in Ss infection by comparing frequencies of Th9 cells at baseline and following antigen-stimulation in infected (INF) with uninfected (UN) control individuals. We demonstrate that Ss infection was associated with elevated frequencies of spontaneously expressed or antigen induced mono and dual functional CD4^+^ Th9 cells. This was further confirmed by elevated levels of IL-9 production in whole blood cultures in INF individuals. The induction of Th9 cells was dependent on IL-10 and TGFβ and was reversible following anti-helmintic chemotherapy.

## Methods

### Ethics statement

All individuals were examined as part of a natural history study protocol approved by Institutional Review Boards of both the National Institutes of Allergy and Infectious Diseases and the National Institute for Research in Tuberculosis (NCT00375583 and NCT00001230), and informed written consent was obtained from all participants.

### Study population

We studied a total of 66 individuals comprising of 43 clinically asymptomatic, Ss infected (hereafter INF) individuals and 23 uninfected, endemic normal (hereafter UN) individuals in Tamil Nadu, South India (Table 1). 28 of the INF individuals were used for in vitro culture and flow cytometry and ELISA while 15 of the INF individuals were used for cytokine neutralization experiments alone. Ss infection was diagnosed by the presence of IgG antibodies to two recombinant antigens—NIE and SsIR by the Luciferase Immunoprecipitation System Assay (LIPS), as described previously [24]. Only those individuals who tested positive by LIPS assay to both antigens were classified as INF. This was further confirmed by specialized stool examination with nutrient agar plate cultures. All individuals were also negative for filarial infection by filarial antigen tests and for other intestinal helminths by stool microscopy. All INF individuals were treated single doses of ivermectin and albendazole and follow-up blood draws were obtained six months later in 15 individuals. All UN individuals were LIPS assay negative, negative for filarial or other intestinal helminths and without any signs or symptoms of infection or disease. There were no differences between the groups in terms of demographics or socio-economic status.

**Table 1:** Demographics of the study population.

	INFECTED (n = 43)	UNINFECTED (n = 23)
**M/F**	21/22	10/13
**Age**	40.18 (18–61)	38 (20–63)
**NIE and SsIR LIPS**	Positive	Negative
**Filarial circulating antigen**	Negative	Negative
**Stool microscopy for other intestinal helminths**	Negative	Negative

### Parasite and control antigen

Saline extracts of *S*. *stercoralis* somatic larval antigens (hereafter SsAg) and recombinant NIE antigen (hereafter NIE) were used for parasite antigens and mycobacterial PPD (Serum Statens Institute, Copenhagen, Denmark) was used as the control antigen. Final concentrations were 10 μg/ml for SsAg, NIE and PPD. Endotoxin levels in the SsAg was < 0.1 EU/ml using the QCL-1000 Chromogenic LAL test kit (BioWhittaker). Phorbol myristoyl acetate (PMA) and ionomycin at concentrations of 12.5 ng/ml and 125 ng/ml (respectively), were used as the positive control stimuli.

### In vitro culture

Whole blood cell cultures were performed to determine the frequencies of intracellular cytokine-producing cells. Briefly, whole blood was diluted 1:1 with RPMI-1640 medium, supplemented with penicillin/streptomycin (100 U/100 mg/ml), L-glutamine (2 mM), and HEPES (10 mM) (all from Invitrogen, San Diego, CA) and placed in 12-well tissue culture plates (Costar, Corning Inc., NY, USA). The cultures were then stimulated with SsAg, NIE, PMA/ionomycin (P/I) or media alone in the presence of the co-stimulatory reagent, CD49d /CD28 (BD Biosciences) at 37°C for 6 or 18 h, for intracellular cytokine staining or ELISA respectively. Fast Immune Brefeldin A Solution (10μg/ml) (BD Biosciences) was added after 2 hours. After 6 hours, whole blood was centrifuged, washed using cold PBS, and then 1x FACS lysing solution (BD Biosciences, San Diego, CA, USA) was added. The cells were fixed using cytofix/cytoperm buffer (BD Biosciences, San Diego, CA, USA), cryopreserved, and stored at -80°C until use. For cytokine neutralization experiments (n = 15), whole blood from INF individuals was cultured in the presence of anti-IL-10 (5μg/ml) or anti-TGFβ (5μg/ml) or isotype control antibody (5μg/ml) (R& D Sytems) for 1 h following which NIE and brefeldin A was added and cultured for a further 23 h.

### Intracellular cytokine staining

The cells were thawed and washed with PBS first and PBS / 1% BSA later and then stained with surface antibodies for 30–60 minutes. Surface antibodies used were CD3, CD4 and CD8 (all from BD Biosciences). The cells were washed and permeabilized with BD Perm/Wash buffer (BD Biosciences) and stained with intracellular cytokines for an additional 30 min before washing and acquisition. Cytokine antibodies used were IL-4, IL-9 and IL-10 (all from BD Pharmingen). Flow cytometry was performed on a FACS Canto II flow cytometer with FACSDiva software v.6 (Becton Dickinson). The lymphocyte gating was set by forward and side scatter and 100,000 gated lymphocyte events were acquired. Data were collected and analyzed using Flow Jo software. All data are depicted as frequency of CD4^**+**^ T cells expressing cytokine(s) or as the mean fluorescence intensity (MFI) of cytokine expression within a particular subset. Mono-functional Th9 cells were defined as CD4^+^ T cells expressing IL-9 alone while dual-functional Th9 cells were those expressing IL-9 plus IL-10 and Th2 cells expressing IL-9 were those that also expressed IL-4. Frequencies following media stimulation are depicted as baseline frequencies while frequencies following stimulation with antigens or P/I are depicted as net frequencies (with baseline subtracted).

### ELISA

Whole blood culture supernatants at 18 h was used for performing IL-9 ELISA. IL-9 was measured using the R&D Systems IL-9 ELISA Duoset kit, according to the manufacturer's instructions.

### Statistical analysis

Data analyses were performed using GraphPad PRISM (GraphPad Software, Inc., San Diego, CA, USA). Geometric means (GM) were used for measurements of central tendency. Statistically significant differences between the two groups were analyzed by Mann-Whitney test and multiple comparisons corrected by Holm’s correction. Statistically significant differences between pre- and post- treatment as well as following cytokine blockade were analyzed by Wilcoxon signed rank test.

## Results

### Ss infection is associated with a spontaneous and an antigen-specific increase in the frequencies of mono- and dual-functional CD4^+^ Th9 cells

**Fig 1:**
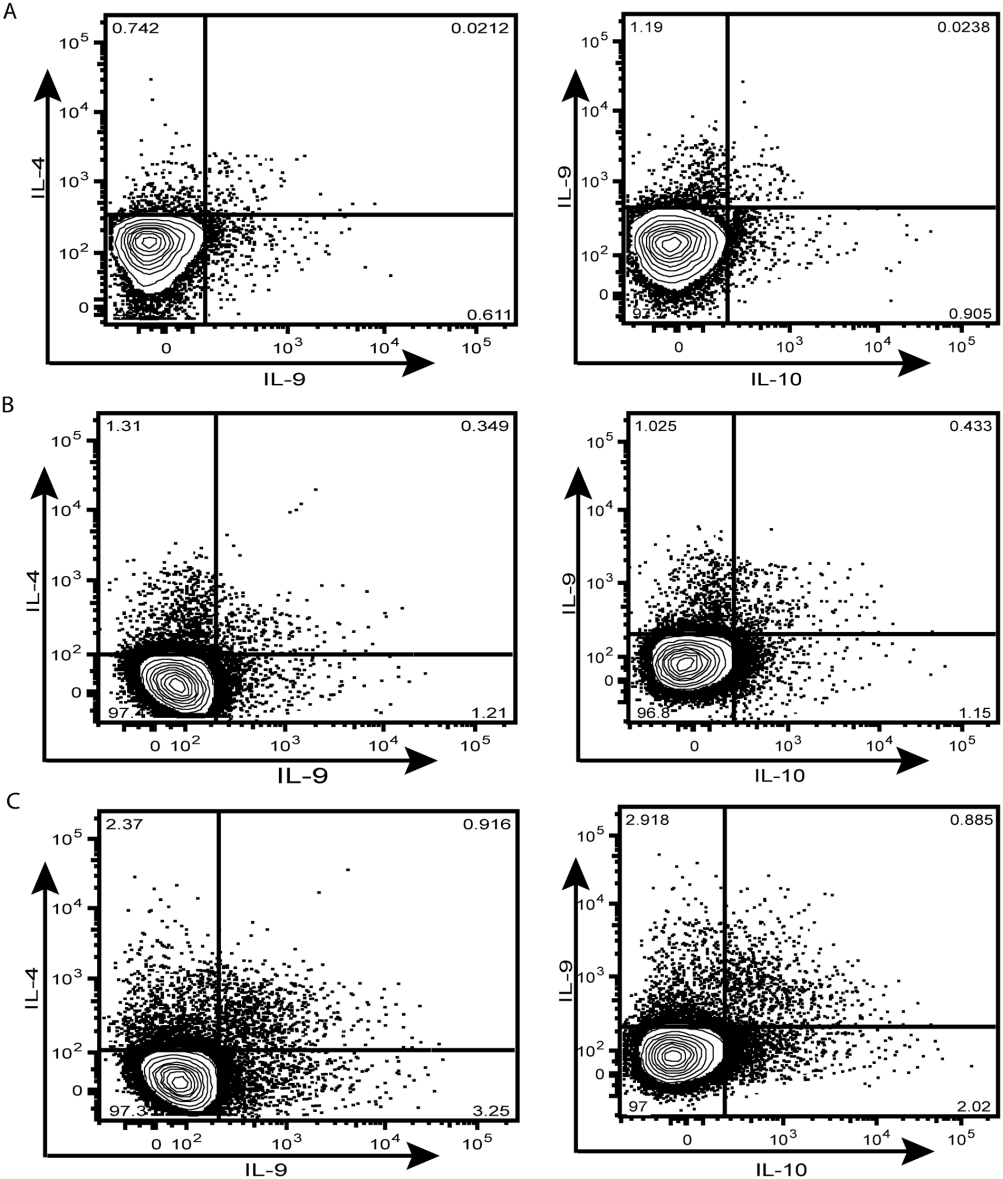
Strongyloides infection is associated with expansion of mono- and dual-functional Th9 cells. Whole blood was cultured with media alone for 6 h and the baseline and antigen-specific frequencies of Th9 cells determined. A representative whole-blood intracellular cytokine assay flow data from a INF individual showing expression of IL-9, IL-4 and IL-10 at baseline (A) and following stimulation with SsAg (B) or P/I (C). The plots shown are gated on CD3^+^CD4^+^ T cells.

**Fig 2:**
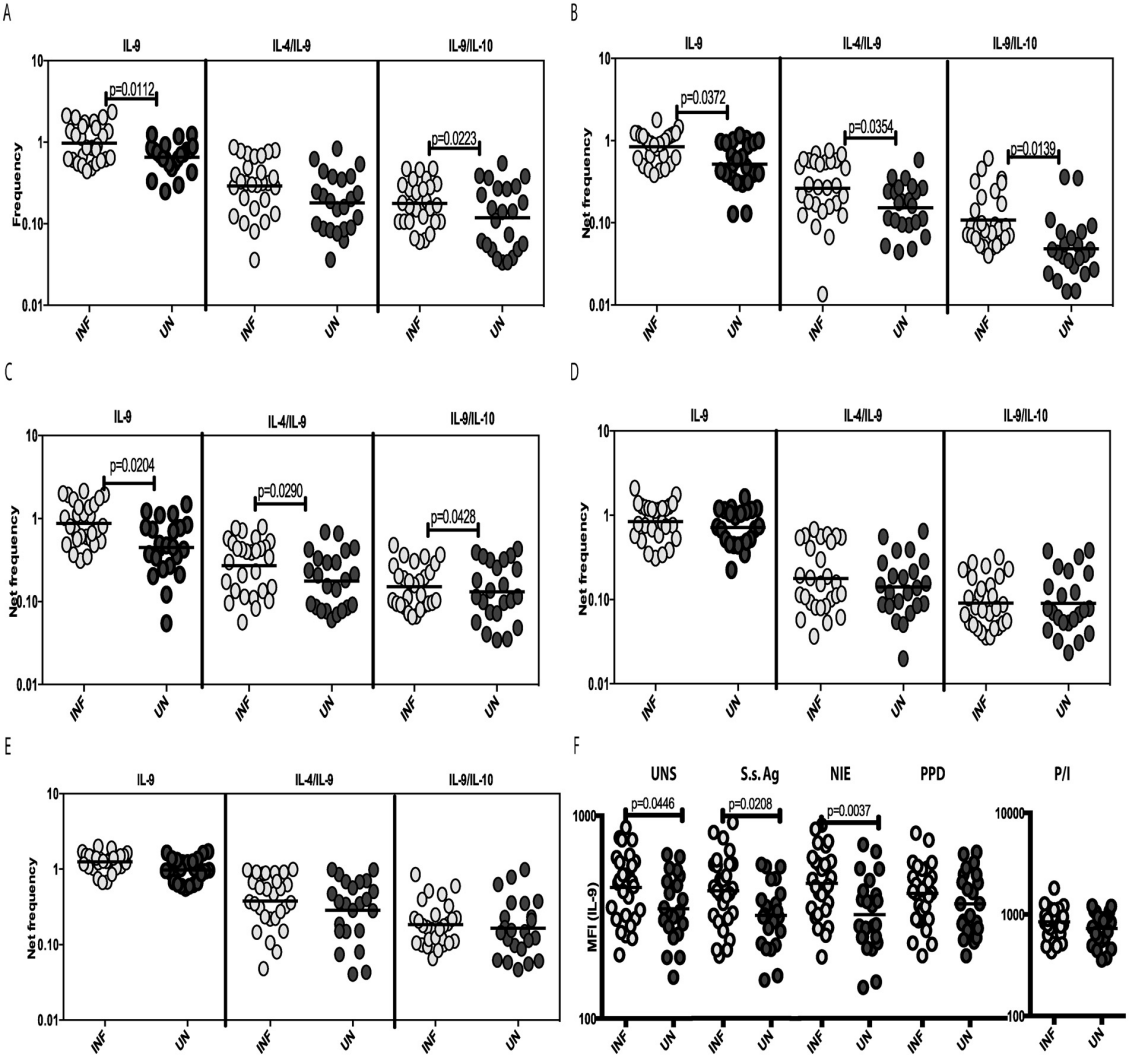
Strongyloides infection is associated with increased spontaneously expressed and antigen-induced frequencies of CD4^+^ mono- and dual-functional Th9 cells. Whole blood was cultured with media alone or with antigens for 6 h and the baseline and antigen-stimulated frequencies of Th9 cells determined. The baseline (A) as well as SsAg (B), NIE (C), PPD (D) and P/I (E) stimulated frequencies of mono- and dual-functional CD4^+^ Th9 cells in INF (n = 28) and UN (n = 23) individuals are shown. The MFI of IL-9 expression on CD4^+^ T cells in INF and UN individuals at baseline and following stimulation are shown (F). Each circle represents a single individual and the bars represent the geometric mean values. Net frequencies were calculated by subtracting baseline frequencies from the antigen-induced frequencies for each individual. *P* values were calculated using the Mann-Whitney test.

To examine the baseline (or steady state) as well as antigen-stimulated expression pattern of Th9 cells in Ss infections, we cultured whole blood from INF and UN individuals with media alone or with SsAg, NIE, PPD or P/I and measured the frequency of CD4^+^ T cells expressing IL-9 or co-expressing IL-9, IL-4 or IL-10. A representative plot is shown in Fig 1. As shown in Fig 2A, the baseline frequency of CD4^+^ T cells expressing IL-9 alone (mono-functional Th9 cells) or the frequency of CD4^+^ T cells co-expressing IL-9/IL-10 (dual-functional Th9 cells) was significantly increased in INF individuals. Similarly, the frequency of mono- and dual-functional Th9 cells was also increased significantly in response to SsAg and NIE stimulation (Fig 2B and 2C). Interestingly, the frequency of Th2 cells expressing IL-9 was also significantly increased in response to antigen stimulation. In contrast, neither PPD (Fig 2D) nor P/I (Fig 2E) induced any significant difference in the frequencies of CD4^+^ Th9 cells between the 2 groups In addition, as shown in Fig 2F, the MFI of IL-9 expression on CD4^+^ T cells was significantly higher in INF individuals at baseline and following SsAg and NIE stimulation. Thus, Ss infection is associated with marked increases in the repertoire of mono- and dual-functional Th9 cells at steady state and following parasite—antigen stimulation as well as with marked enhancement of IL-9 expression on a per cell basis in CD4^+^ T cells.

### IL-10 and TGFβ mediate the expansion of mono- and dual-functional Th9 in Ss infections

**Fig 3:**
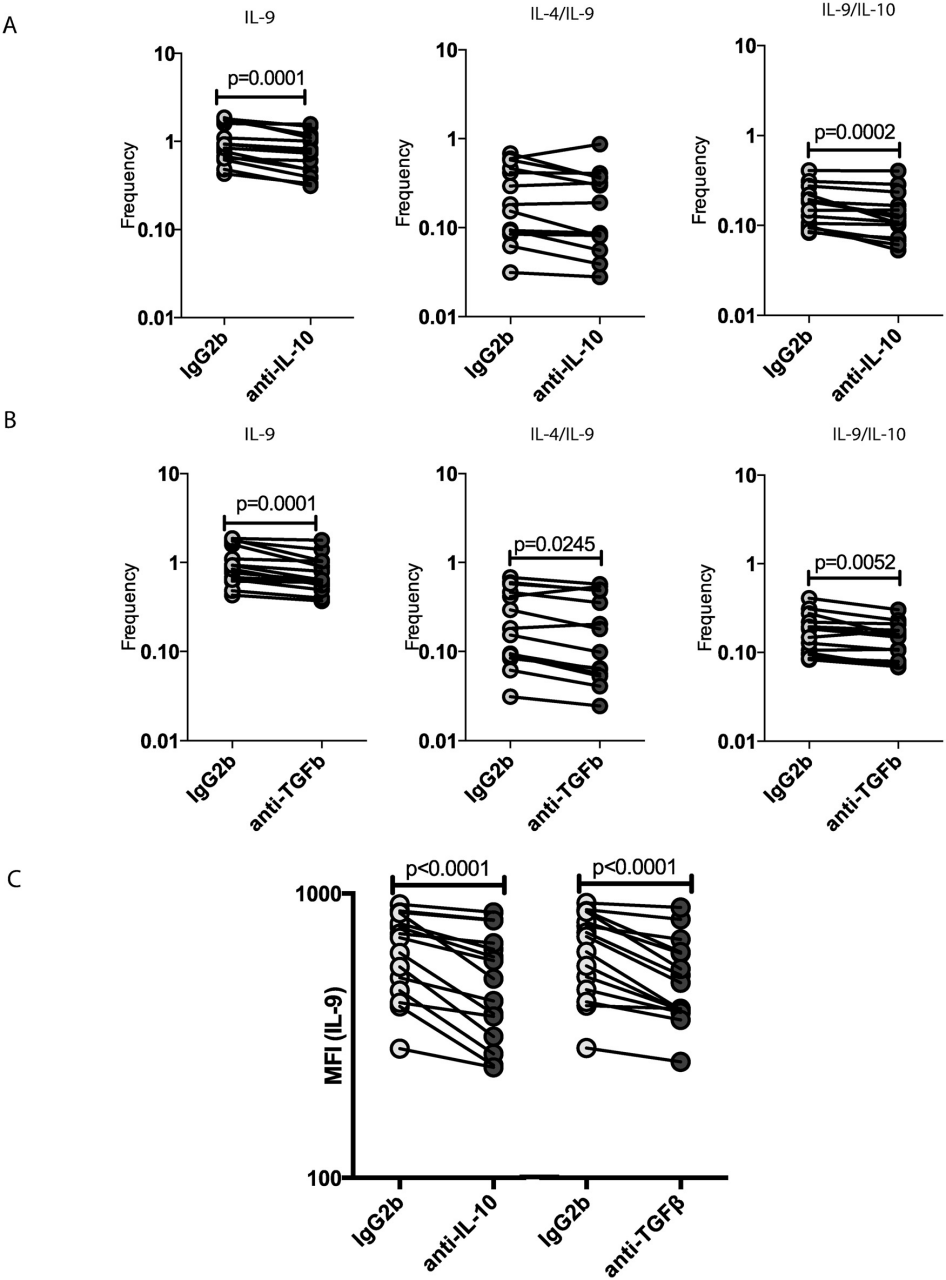
IL-10 and TGFβ mediate the expansion of mono- and dual-functional Th9 cells in Strongyloides infections. (A) IL-10 neutralization (with anti-IL-10 antibody) significantly diminishes the frequencies of mono- functional or dual-functional CD4^+^ Th9 cells following stimulation with NIE in a subset of INF individuals (n = 15). (B) TGFβ neutralization (with anti-TGFβ antibody) significantly diminishes the frequencies of mono- functional or dual-functional CD4^+^ Th9 cells following stimulation with NIE in a subset of INF individuals (n = 15). (C) Both IL-10 and TGFβ neutralization significantly reduce the MFI of IL-9 expression on CD4^+^ T cells following stimulation with NIE in a subset of INF individuals (n = 15). Antigen-stimulated frequencies are shown as net frequencies with the baseline levels subtracted. Each line represents a single individual. P values were calculated using the Wilcoxon signed rank test.

To determine the role of IL-10 and TGFβ in the modulation of Th9 cells in INF, we measured the frequency of these cells following stimulation with the parasite antigen NIE in the presence or absence of anti-IL-10 or anti- TGFβ neutralizing antibody in INF individuals (n = 15). As shown in Fig 3A, IL-10 neutralization resulted in significantly decreased frequencies of mono-functional and dual-functional Th9 cells in INF individuals. Similarly, as shown in Fig 3B, TGFβ neutralization also resulted in significantly decreased the frequencies of mono-functional and dual-functional Th9 cells in INF individuals. Interestingly, only TGFβ but not IL-10 blockade significantly decreased the frequency of Th2 cells expressing IL-9. In addition, as shown in Fig 3C, the MFI of IL-9 expression on CD4^+^ T cells was significantly diminished following both IL-10 and TGFβ blockade, indicating that the cytokine expression on a per cell basis is also modulated by these regulatory cytokines. Thus, IL-10 and TGFβ play important roles in the antigen-induced expansion of Th9 cells in Ss infections.

### Treatment of Ss infection results in significantly reduced frequencies of antigen-stimulated Th9 cells

**Fig 4:**
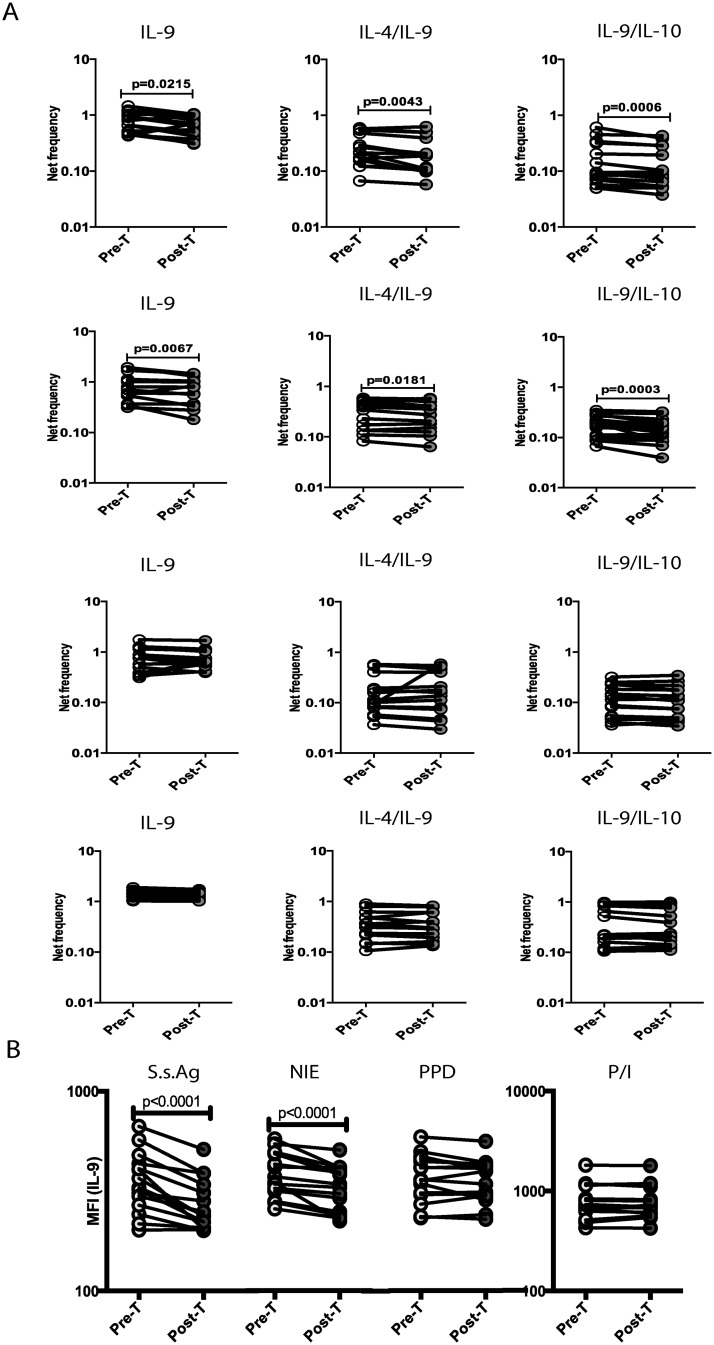
Treatment of Strongyloides infection is associated with diminished frequencies of antigen-specific Th9 cells. (A) The frequencies of Th9 cells following stimulation with SsAg, NIE, PPD and P/I before and after treatment with a standard dose of ivermectin and albendazole in a subset of INF individuals (n = 15). (B) The MFI of IL-9 expression following stimulation with SsAg, NIE, PPD and P/I before and after treatment with a standard dose of ivermectin and albendazole in a subset of INF individuals (n = 15). Antigen-stimulated frequencies are shown as net frequencies with the baseline levels subtracted. Each line represents a single individual. P values were calculated using the Wilcoxon signed rank test.

To determine the role of active infection in the regulation of mono- and dual-functional Th9 cells in Ss infections, we measured the Th9 response in a subset of INF individuals (n = 15), who had been treated with anti-helmintic chemotherapy six months earlier. As shown in Fig 4A, treatment of Ss infection resulted in significantly reduced frequencies of mono- and dual-functional Th9 cells in response to SsAg or NIE stimulation but not in response to PPD or P/I. In addition, as shown in Fig 4B, the MFI of IL-9 expression on CD4^+^ T cells is also significantly decreased in response to SsAg or NIE stimulation but not in response to PPD or P/I following treatment. Thus, the antigen-driven expansion of CD4^+^ Th9 cells in Ss infection is reversible (for the most part) following treatment of infection.

### Ss infection is associated with increased baseline and antigen-specific production of IL-9, which is reversible following treatment

**Fig 5:**
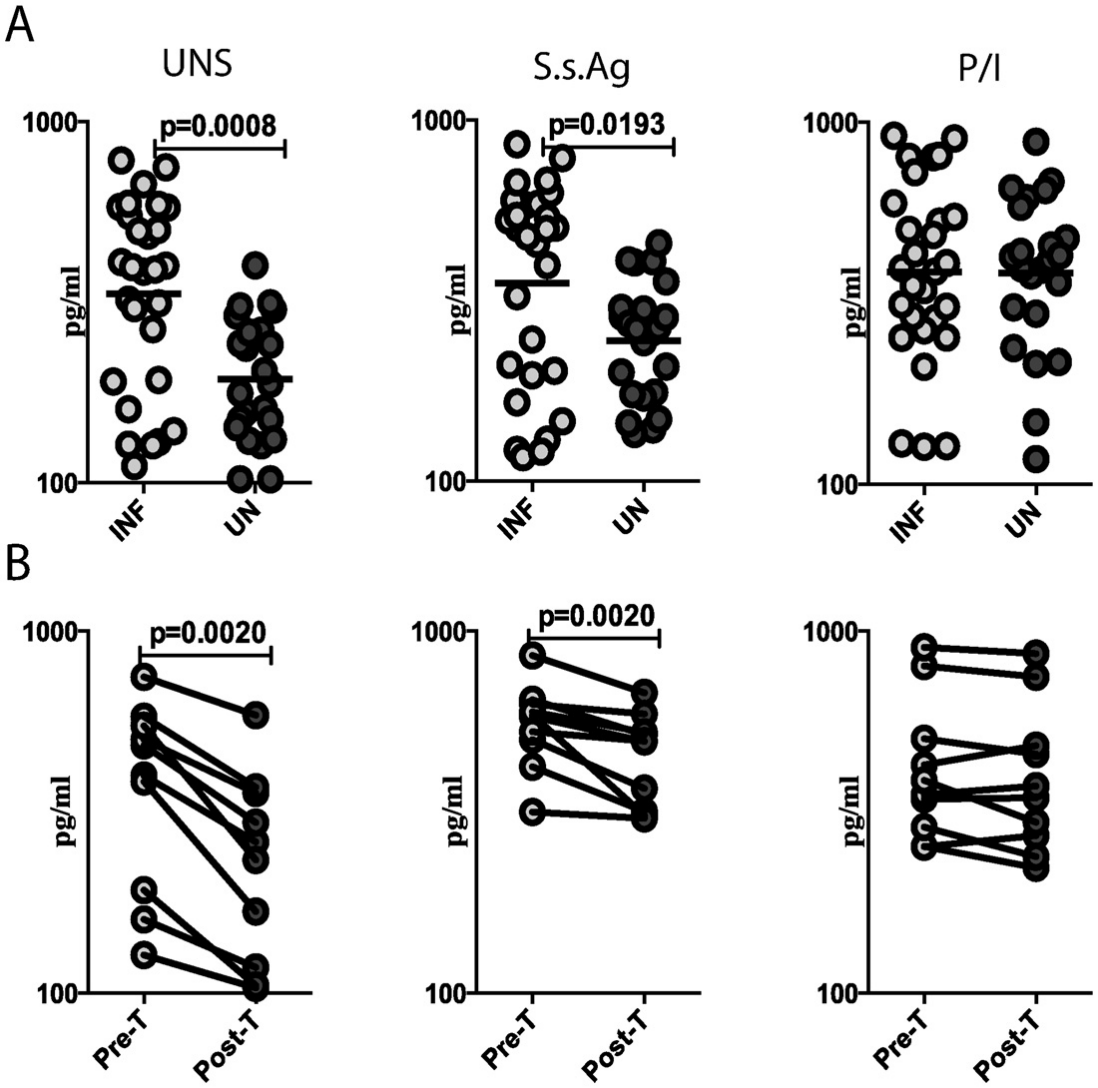
Strongyloides infection is associated with increased spontaneously produced and antigen-induced production of IL-9. Whole blood was cultured with media alone or with Ss Ag or P/I for 18 h and the baseline and antigen-stimulated levels of IL-9 determined by ELISA. (A) The baseline as well as SsAg and P/I stimulated levels of IL-9 in INF (n = 28) and UN (n = 23) individuals are shown. Each circle represents a single individual and the bars represent the geometric mean values. *P* values were calculated using the Mann-Whitney test. (B) The IL-9 levels following stimulation with media alone, SsAg and P/I before and after treatment with a standard dose of ivermectin and albendazole in a subset of INF individuals (n = 15). Each line represents a single individual. P values were calculated using the Wilcoxon signed rank test.

To examine the baseline (or steady state) as well as antigen-stimulated production of IL-9 in Ss infections, we cultured whole blood from INF and UN individuals with media alone or with Ss Ag or P/I and measured the levels of IL-9 production in the supernatants at 18 h by ELISA. As shown in Fig 5A, the production of IL-9 at baseline and following Ss Ag stimulation was significantly increased in INF compared to UN individuals. Moreover, as shown in Fig 5B, this elevated production of IL-9 was significantly decreased in INF individuals following treatment. Thus, Ss associated enhancement of Th9 cell frequencies is reflected by a concomitant increase in IL-9 cytokine levels.

## Discussion

Although IL-9 is known to be expressed by several types of immune cells, IL-9 secreting CD4^+^ T cells are a predominant source of IL-9 in allergic inflammation and anti-parasite immunity [6,7]. Th9 cells in human diseases are known to contribute to both protective immune responses and pathological responses leading to immune mediated pathology [7]. The role of IL-9 in helminth infection was first suggested by animal studies showing that IL-9 transgenic mice infected with *Trichuris muris* or *Trichinella spiralis* had an increased Th2 response and faster expulsion of the parasite from the intestine [15,16,17]. While classically considered a Th2 cytokine, IL-9 has now been shown to produced by a distinct subset of CD4^+^ T cells that express IL-9 with or without IL-10 but in the absence of IL-4 [4,5]. Whether the source of IL-9 ultimately matters in the context of resistance to infection is still not clear. However, a more recent study has clearly shown that IL-9 is produced early during *Nippostrongylus brasiliensis* infection of mice by a non-Th2 CD4^+^ T cell subset and that its production from this subset is sufficient for host protection against worm infection [25].

Our study on the regulation of Th9 cells in Ss infection reveals the presence of increased frequencies of Th9 cells (IL-9 single and IL-9/IL-10 double expressing) at baseline in INF individuals compared to UN individuals. The increased frequency is further augmented upon stimulation with two different parasite antigens, indicating that the Th9 cells are parasite specific and respond to recall stimulation. In addition to classical Th9 cells responding to antigen-stimulation, we also observed increased frequencies of Th2 cells expressing IL-9 (IL-4/IL-9 co-expressing), indicating that other CD4^+^ T cell subsets also respond to Ss infection with the capacity to produce more IL-9. This confirms our previous data demonstrating elevated Th2 responses in Ss infection and its reversal following anti-helmintic therapy [23]. This study also confirms data from the animal models of helminth infection showing expansion of IL-9 expressing CD4^+^ T cells [25]. Although the kinetics of Th9 induction compared to the induction of Th2 cells cannot be determined from this study, our data clearly reveal that Th9 responsiveness (that is separate from Th2 responses) is a major feature of the antigen-specific T cell response in this human helminth infection. Moreover, our data also indicate that the enhanced frequencies of Th9 cells is reflected in elevated expression of IL-9 on a per cell basis in Ss infections. Finally, we also confirm that Ss infections are associated with elevated levels of antigen-stimulated IL-9 levels by using ELISA. Thus, by using two different methodologies, we verify the important association of enhanced Th9 responses in Ss infections and its reversibility following treatment. While we have demonstrated statistically significant changes in Th9 responses and have verified this with actual cytokine levels, we do acknowledge the fact that these changes are small in magnitude.

Previous studies have shown that IL-2, IL-4 and TGFβ are the primary factors that drive differentiation and expansion of Th9 cells [7]. Moreover, we have previously shown that Th2 and regulatory cytokines are increased in Ss infection [23]. Therefore, we wanted to examine the role of IL-10 and TGFβ in the induction of Th9 responses. In this study, we observed that IL-10 in addition to TGFβ also appears to play an important role in regulating the expansion of Th9 cells in Ss infection. The exact mechanism by which IL-10 modulates the expansion of Th9 cells is yet to be determined. Finally, we demonstrate an important role for the persistence of antigen in the maintenance of the Th9 response. Our data clearly illustrate that anti-helmintic treatment results in significantly depressed Th9 responses, implying that sustained, chronic infection is a major driver of Th9 maintenance. We postulate that the induction of IL-10 and TGFβ in chronic infection upregulates Th9 expression that is reversible upon anti-helmintic treatment.

IL-9 is known to act on a wide variety of cells and perform multiple functions [26]. For example, IL-9 stimulates the growth, proliferation and survival of T cells [27,28], enhances the production of IgE from B cells [29], promotes the proliferation and differentiation of mast cells and hematopoietic progenitors and induces secretion of mucus and chemokines by mucosal epithelial cells [30]. Therefore, IL-9 has the ability to act as a critical cytokine in mucosal infections especially helminth infections and indeed, has been shown to be crucial driver of the host immune response against these infections in animal models. Our study extends these findings to human gastrointestinal helminth infection and shows that non-Th2 Th9 responses are a major feature of Ss infection, responses that likely require IL-10 and TGFβ for their maintenance.
